# The emerging plasma biomarker Dickkopf-3 (DKK3) and its association with renal and cardiovascular disease in the general population

**DOI:** 10.1038/s41598-021-88107-9

**Published:** 2021-04-21

**Authors:** Arnold Piek, Leonie Smit, Navin Suthahar, Stephan J. L. Bakker, Rudolf A. de Boer, Herman H. W. Silljé

**Affiliations:** 1 Department of Cardiology, University Medical Center Groningen, University of Groningen, Hanzeplein 1, Groningen, 9713 GZ The Netherlands; 2 Department of Internal Medicine, University Medical Center Groningen, University of Groningen, Hanzeplein 1, Groningen, 9713 GZ The Netherlands; 3 P.O. Box 30.001, 9700 RB Groningen, The Netherlands

**Keywords:** Cardiology, Medical research, Biomarkers

## Abstract

Dickkopf-3 (DKK3) is an emerging biomarker for cardiovascular disease (CVD) and chronic kidney disease (CKD). Herein, baseline DKK3 plasma levels were measured in 8420 subjects from the Prevention of Renal and Vascular ENd-stage Disease (PREVEND) cohort, a large general population cohort, using enzyme-linked immunosorbent assays. Associations with clinical variables and outcomes were analysed. Median DKK3 level was 32.8 ng/ml (28.0–39.0). In multivariable linear regression analysis, the strongest correlates for plasma DKK3 were age, body mass index and estimated glomerular filtration rate (eGFR). At baseline, 564 (6.7%) subjects had CVD (defined as a myocardial infarction and/or cerebrovascular accident) and 1361 (16.2%) subjects had CKD (defined as eGFR < 60 ml/min/1.73m^2^ and/or urinary albumin excretion (UAE) > 30 mg/24 h). Of subjects with known CVD and CKD follow-up status (respectively 7828 and 5548), 669 (8.5%) developed CVD and 951 (17.1%) developed CKD (median follow-up respectively 12.5 and 10.2 years). Crude logistic regression analysis revealed that DKK3 levels were associated with prevalent CVD (Odds ratio: 2.14 **[**1.76–2.61] per DKK3 doubling, P < 0.001) and CKD (Odds ratio: 1.84 [1.59–2.13] per DKK3 doubling, P < 0.001). In crude Cox proportional hazard regression analysis, higher DKK3 levels were associated with higher risk for new-onset CVD (Hazard ratio: 1.47 [1.13–1.91] per DKK3 doubling, P = 0.004) and CKD (Hazard ratio: 1.45, [1.25–1.69] per DKK3 doubling, P < 0.001). However, these associations remained no longer significant after correction for common clinical variables and risk factors, though independently predicted for new-onset CKD in a subgroup of subjects with the lowest UAE values. Together, DKK3 plasma levels are associated with cardiovascular risk factors, but are generally not independently associated with prevalent and new-onset CVD and CKD and only predicted for new-onset CKD in those subjects with the lowest UAE values.

## Introduction

With the ageing population, diseases associated with life style including amongst others cardiovascular disease (CVD) and chronic kidney disease (CKD) are expected to become more prevalent. In Western countries CVD is one of the major causes of death, and accounts for about 18 million deaths worldwide annually^1^. CKD is also a prevalent disease, with a worldwide estimated prevalence of 11–13%^2^. Additional tools to identify prevalent cases of CVD and CKD, but also to identify cases at risk for future disease, could improve and guide current treatment and prevention strategies, thereby reducing morbidity and mortality for many individuals. Plasma biomarkers hold great potential to fulfill such tasks.

Dickkopf-3 (DKK3) is a secreted circulatory protein and is a member of the Dickkopf family^3^. DKKs, especially DKK 1, 2 and 4, are predominantly known for their inhibitory effect on the Wnt signaling pathway, which is involved in embryonic development, carcinogenesis, and also in cardiovascular disease^3,4^. The role of DKK3 in Wnt pathway signaling is, however, still elusive. Nevertheless, a role for DKK3 in CVD including HF and renal disease was suggested. In particular, studies with DKK3 transgenic and knock out mouse models have suggested a protective role of DKK3 in the heart, but a detrimental effect in the kidney^5–10^. Its function in atherosclerosis has been investigated in apolipoprotein E-deficient (ApoE^−/−^) mice with mixed results^11–13^.

Recently, the protein DKK3 gained attention as an emerging biomarker for cardiovascular and renal diseases. In a cohort derived from the general population, plasma levels of DKK3 were inversely associated with atherosclerosis development^13^. Besides, urinary DKK3 was also studied and proposed to be a renal tubular stress marker^10^. In patients with pre-existing CKD, elevated urinary DKK3 levels at baseline improved existing risk prediction models for loss of kidney function^14^. Moreover, preoperative DKK3 urine levels in patients undergoing cardiac surgery were predictive for post-operative acute kidney injury (AKI) and increased risk for long-term loss of kidney function^15^. Finally, in a large cohort of heart failure (HF) patients, plasma DKK3 levels were associated with several cardiovascular risk factors, including kidney function, atrial fibrillation, body mass index (BMI) and blood pressure^16^.

Herein, we measured DKK3 plasma levels in 8420 subjects of the Prevention of Renal and Vascular ENd-stage Disease (PREVEND) cohort, which is a cohort with community-dwelling subjects designed to study cardiovascular risk factors and renal function, as well as prevalent and new-onset CVD and CKD. The associations of DKK3 plasma levels with cardiovascular risk factors, and with both prevalent and new-onset CVD and CKD were determined.

## Results

### Study population and baseline characteristics

**Figure 1:**
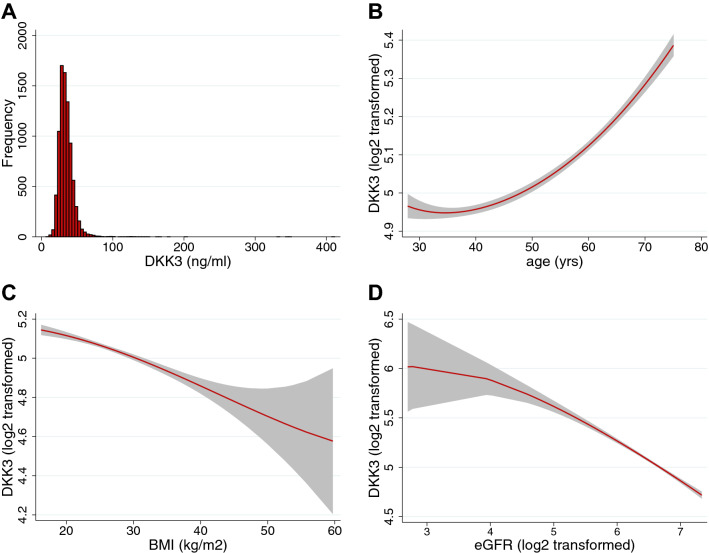
DKK3 plasma concentrations and their relationship with age, BMI and eGFR. (**A**) Frequency distribution of DKK3 plasma levels in the PREVEND cohort. (**B**–**D**) Two-way fractional polynomial regression plots (in red) with 95% confidence intervals (CI, in grey) showing the relationship of plasma Dickopf-3 (DKK3) concentrations with (**B**) age, (**C**) body mass index (BMI) and (**D**) estimated glomerular filtration rate (eGFR). DKK3 and eGFR values are log2 transformed.

DKK3 levels were determined in plasma samples from 8420 subjects included in the PREVEND cohort. Mean age of the cohort was 49.3 ± 12.6, and the distribution of males and females was nearly equal (50.2% vs. 49.8%, respectively). Median DKK3 level was 32.8 ng/ml (28.0–39.0) and showed a skewed distribution (Fig. 1A). Baseline characteristics according to DKK3 quintiles are presented in Table 1. Subjects with higher plasma levels of DKK3 were generally older, more often of the male sex and had a lower body mass index (BMI) (Table 1). Also, with increasing DKK3 quintiles, a higher prevalence of hypertension, chronic kidney disease (CKD) and cardiovascular disease (CVD) was observed (Table 1). Finally, subjects in the higher DKK3 quintiles had higher cholesterol levels and higher urinary albumin excretion (UAE), whilst estimated glomerular filtration rate (eGFR) and levels of C-reactive protein (CRP) were lower (Table 1). Together, higher DKK3 levels are associated with increased presence of cardiovascular risk factors, particularly age, male sex, history of hypertension, CKD, CVD and higher cholesterol levels.

**Table 1:** Baseline characteristics according to DKK3 quintiles.

	DKK3, ng/ml										P for Trend
	Q1: ˂ 26.9		Q2: 26.9–30.8		Q3: 30.8–35.0		Q4: 35.0–40.7		Q5: > 40.7		
	(n = 1684)		(n = 1684)		(n = 1684)		(n = 1684)		(n = 1684)		
**Clinical characteristics**											
Age (years)	44 (10)		47 (12)		49 (12)		52 (13)		55 (13)		< 0.001
Male sex, n (%)	803 (48)		817 (49)		846 (50)		846 (51)		870 (52)		0.007
SBP (mmHg)	127 (18)		128 (19)		128 (19.7)		130 (22)		132 (23)		< 0.001
BMI, mean (kg/m^2^)	27.2 (4.7)		26.3 (4.3)		25.9 (4.2)		25.6 (3.9)		25.5 (3.8)		< 0.001
**Medical history, n (%)**											
Smoking (last year)	638 (38)		647 (39)		636 (38)		643 (39)		610 (37)		0.414
Diabetes	67 (4)		63 (4)		55 (3)		67 (4)		77 (5)		0.310
Hypertension	398 (24)		405 (24)		415 (25)		511 (31)		552 (33)		< 0.001
CKD	218 (14)		204 (13)		264 (17)		301 (19)		374 (23)		< 0.001
CVD	56 (3)		89 (6)		124 (8)		130 (8)		165 (10)		< 0.001
**Laboratory measurements**											
Glucose (mmol/L)	4.8 (4.4–5.2)		4.7 (4.3–5.2)		4.7 (4.3–5.1)		4.7 (4.3–5.1)		4.7 (4.3–5.1)		< 0.001
Cholesterol (mmol/L)	5.6 (1.2)		5.6 (1.1)		5.6 (1.1)		5.7 (1.1)		5.7 (1.2)		0.001
eGFR (mL/min/1.73m^2^)	103 (92–111)		100 (89–110)		97 (86–107)		94 (81–105)		88 (74–100)		< 0.001
24 h UAE (mg/24 h)	9.0 (6.3–16.1)		9.0 (6.3–15.4)		9.3 (6.3–17.4)		10.0 (6.4–19.4)		10.1 (6.4–21.9)		< 0.001
CRP (mg/L)	1.7 (0.7–4.0)		1.3 (0.6–3.1)		1.2 (0.5–2.7)		1.1 (0.5–2.7)		1.2 (0.5–2.7)		< 0.001

Continuous variables are represented as mean ± standard deviation (SD) for normally distributed data and as median (interquartile range, IQR) for non-normally distributed data. Categorical variables are represented as n (%).

*BMI* Body mass index, *CKD* Chronic kidney disease, *CRP* C-reactive protein, *CVD* Cardiovascular disease, *DKK3* Dickkopf-3, *eGFR* Estimated glomerular filtration rate, *SBP* Systolic blood pressure, *UAE* Urinary albumin excretion.

### Factors associated with DKK3

Next, linear regression analyses were performed, and the results are shown in Table 2. Herein, strong correlates for DKK3 were age (sβ: 0.281, P ˂ 0.001), eGFR (sβ: − 0.300, P ˂ 0.001) and BMI (sβ: − 0.132, P ˂ 0.001). Assumptions of normally distributed and proportional residuals were upheld. The relationships of DKK3 with age, BMI and eGFR were further explored using fractional polynomial regression analysis and are visualized in Fig. 1B–D, showing that generally DKK3 levels are higher in older subjects and lower in subjects with higher BMI and as well in subjects with lower eGFR. Next, using multivariable linear regression analysis, age, eGFR, BMI, CRP, glucose and sex were identified as factors independently associated with DKK3 levels (Table 3). Assumptions of normally distributed and proportional residuals were upheld and no multicollinearity was observed in this multivariable model. Together, these factors explained approximately 18% of the variation in DKK3 levels.

**Table 2:** Relationship between DKK3 levels and baseline characteristics.

	Sβ	R^2^	P-value
**Clinical characteristics**			
Age	0.281	0.079	˂ 0.001
Female sex	− 0.038	0.001	˂ 0.001
SBP	0.076	0.006	˂ 0.001
BMI	− 0.132	0.017	˂ 0.001
**Medical history**			
Smoking (last year)	− 0.023	˂ 0.001	0.037
Diabetes	0.004	0.000	0.705
Hypertension	0.073	0.005	˂ 0.001
**Laboratory measurements**			
Glucose^†^	− 0.043	0.002	˂ 0.001
Cholesterol	0.035	0.001	0.001
eGFR^†^	− 0.300	0.090	˂ 0.001
24 h UAE^†^	0.071	0.005	˂ 0.001
CRP^†^	0.092	0.008	˂ 0.001

*Sβ* standardized beta coefficient. Other abbreviations as in Table 1.

^†^Log2-transformed.

**Table 3:** Multivariable model including DKK3-associated factors.

DKK3†	Β ± SE	Sβ	P-value
Age	0.008 ± 0.000	0.246	˂ 0.001
eGFR^†^	− 0.290 ± 0.018	− 0.221	˂ 0.001
BMI	− 0.015 ± 0.001	− 0.162	˂ 0.001
CRP^†^	− 0.029 ± 0.003	− 0.128	˂ 0.001
Glucose^†^	− 0.122 ± 0.017	− 0.084	˂ 0.001
Female sex	− 0.020 ± 0.008	− 0.026	0.014

*Sβ* standardized beta coefficient. Other abbreviations as in Table 1. R^2^ = 0.175. N = 7460. ^†^Log2-transformed.

### DKK3 and cardiovascular disease

Next, we investigated the association of DKK3 with prevalent and new-onset CVD. Prevalent cardiovascular disease (CVD) was defined as a subject-reported positive history of myocardial infarction and/or cerebrovascular accident. At baseline, 564 subjects (7.0%) out of 8073 subjects with available CVD-status had prevalent CVD. Logistic regression analysis revealed that DKK3 was positively associated with prevalent CVD (OR: 2.14 [1.76–2.61] per DKK3 doubling P < 0.001) and this association remained significant after age-adjustment (OR: 1.35 [1.08–1.70] per DKK3 doubling, P = 0.009), and after adjustment for age and sex (OR: 1.34 [1.07–1.69] per DKK3 doubling, P = 0.010) (Table 4). After adjusting for the multivariable model (model as presented in Table 3) this association remained no longer significant (OR: 1.22 [0.94–1.59] per DKK3 doubling, P = 0.126) (Table 4) and the same was observed after adjustment for the clinical model (OR: 1.15 [0.90–1.49] per DKK3 doubling, P < 0.261) (Table 4). In these models, DKK3 showed no significant interactions with age, sex, BMI, CRP or eGFR.

**Table 4:** DKK3: Prevalent and new-onset CVD.

	Prevalent CVD			New-onset CVD		
	Cases, n (%)	Odds ratio (95% CI)	P-value	Cases, n (%)	Hazard ratio (95% CI)	P-value
DKK3^†^	564 (7.0)	2.14 (1.76–2.61)	< 0.001	669 (8.5)	1.47 (1.13–1.91)	0.004
DKK3^†^, age	564 (7.0)	1.35 (1.08–1.70)	0.009	669 (8.5)	0.76 (0.55–1.06)	0.103
DKK3^†^, age, sex	564 (7.0)	1.34 (1.07–1.69)	0.010	N.A	N.A	N.A
**DKK3**^**†**^**, models**						
Multivariable model	504 (7.0)	1.22 (0.94–1.59)	0.126	N.A	N.A	N.A
Clinical model	523 (7.0)	1.14 (0.89–1.47)	0.309	N.A	N.A	N.A

*CI* Confidence Interval. *CVD* Cardiovascular disease. *DKK3* Dickkopf-3. Multivariable model as presented in Table 3. Clinical model includes age, sex, body mass index, systolic blood pressure, glucose^†^, cholesterol and estimated glomerular filtration rate^†^. Prevalent CVD: N = 8073 for DKK3 crude and age-adjusted, N = 7250 for DKK3 + multivariable model, N = 7480 DKK3 + clinical model. For new-onset CVD: N = 7828. ^†^Log2-transformed.

To investigate the predictive value of DKK3 for new-onset CVD, Cox proportional hazards regression analysis was performed. New-onset CVD was defined as having an event of myocardial infarction, acute or subacute ischemic heart disease, subarachnoid or intracerebral hemorrhage or ischemic stroke, or the need to undergo coronary artery bypass grafting (CABG) or percutaneous coronary intervention (PCI) during the follow-up period. During a median follow-up of 12.5 years, 669 (8.5%) out of 7828 subjects with known follow-up status developed CVD. Again, DKK3 was univariably associated with the risk of new-onset CVD (HR: 1.47 [1.13–1.91] per DKK3 doubling, P = 0.004) (Table 4). After adjusting for age, no significant association between DKK3 plasma levels and new-onset CVD remained (HR: 0.76 [0.55–1.06] per DKK3 doubling, P = 0.103) (Table 4). Fine-Gray competing risk regression models with death as a competing risk were used to correct for fatal-events during follow-up and similar results were obtained (Supplemental Table S1). The same applied for Cox proportional hazards regression analysis with subgroups based on baseline eGFR tertiles and baseline UAE tertiles (Supplemental Tables S2 and S3). Assumptions for proportional hazards were upheld and no significant interaction with age was observed.

### DKK3 and chronic kidney disease

Finally, the association between DKK3 and prevalent and new-onset CKD was analyzed. Prevalent and new-onset chronic kidney disease (CKD) were, according to the KDIGO criteria, defined as an eGFR ˂ 60 ml/min/1.73m^2^ or a UAE > 30 mg/24 h at baseline or during follow-up, respectively^20^. At baseline, 1361 subjects (17.1%) out of 7946 subjects with available CKD-status had CKD. Logistic regression analysis showed that DKK3 levels were positively associated with prevalent CKD (OR: 1.84 [1.59–2.13] per DKK3 doubling, P < 0.001) (Table 5). After age-adjustment, this association was no longer significant (OR: 1.06 [0.90–1.25] per DKK3 doubling, P = 0.472) (Table 5). A significant interaction was observed between DKK3 and age (OR: 1.03 [1.01–1.04], P < 0.001).

**Table 5:** DKK3: Prevalent and new-onset CKD.

	Prevalent CKD			New-onset CKD		
	Cases, n (%)	Odds ratio (95% CI)	P-value	Cases, n (%)	Hazard ratio (95% CI)	P-value
DKK3^†^	1361 (17.2)	1.84 (1.59–2.13)	< 0.001	951 (17.1)	1.45 (1.25–1.69)	<0.001
DKK3^†^, age	1361 (17.2)	1.06 (0.90–1.25)	0.472	951 (17.1)	0.91 (0.76–1.09)	0.306
DKK3^†^, age, sex	N.A	N.A	N.A	N.A	N.A	N.A
**DKK3**^**†**^**, models**						
Multivariable model	N.A	N.A	N.A	N.A	N.A	N.A
Clinical model	N.A	N.A	N.A	N.A	N.A	N.A

*CI* Confidence Interval, *CKD* Chronic kidney disease, *DKK3* Dickkopf-3. Multivariable model as presented in Table 3. Clinical model includes age, sex, body mass index, systolic blood pressure, glucose^†^ and cholesterol. Prevalent CKD: N = 7946. New onset CKD: N = 5548. ^†^Log2-transformed.

During a median follow-up of 10.2 years, amongst 5548 subjects with known follow-up status, 951 (17.1%) subjects developed CKD. Again, in crude Cox proportional hazards regression analysis, DKK3 levels were significantly associated with new-onset CKD (HR: 1.68 [1.38–2.05] per DKK3 doubling, P < 0.001) (Table 5), but after age-adjustment this association was no longer significant (HR: 1.03 [0.80–1.31] per DKK3 doubling, P = 0.832) (Table 5). When corrected for fatal events during follow-up, using Fine-Gray competing risk regression models with death as a competing risk, similar results were obtained (Supplemental Table S4). Also, Cox proportional hazards regression analysis for subgroups based on baseline eGFR tertiles produced similar results (Supplemental Table S5). However, in subgroups created based on baseline UAE tertiles, for subjects in the lower UAE tertile (UAE < 6.6 mg/24 h) DKK3 plasma levels were significantly associated with new-onset CKD, also after correction for age, sex and after correction for the multivariable model or the clinical model (Supplemental Table S6). The latter was not true for the middle and upper UAE tertile subgroups. Assumptions of proportional hazards were upheld for all these analyses and no significant interactions were observed with age, sex, BMI, CRP or eGFR.

## Discussion

In this study, we investigated the associations of Dickkopf-3 (DKK3) plasma levels with cardiovascular risk factors, cardiovascular disease (CVD) and chronic kidney disease (CKD) in the Prevention of Renal and Vascular ENd-stage Disease (PREVEND) cohort, which is a cohort with community-dwelling subjects designed to study cardiovascular risk factors and renal function, as well as both prevalent and new-onset CVD and CKD^17^. Our data show that DKK3 plasma levels are associated with cardiovascular risk factors, including age, male sex, body mass index (BMI) and glucose levels. Although univariably DKK3 is significantly associated with both prevalent and new-onset CVD and CKD, after correction for common clinical variables this association remained no longer significant. Only in subjects with the lowest UAE values, as based on UAE tertiles, plasma DKK3 independently predicted for new-onset CKD. Thus, in this general population cohort as a whole, DKK3 plasma levels do not independently predict for new-onset CVD and CKD.

Previously, *urinary* DKK3 levels were investigated in relation to renal function and renal diseases. Firstly, in patients with CKD, increased levels of urinary DKK3 at baseline were independently predictive for increased risk of future loss of kidney function^14^. Secondly, in patients undergoing cardiac surgery, increased preoperative levels of urinary DKK3 were predictive for the occurrence of postoperative acute kidney injury (AKI) and associated with an increased risk for decline in kidney function in the long-term^15^. Together, since DKK3 is involved in renal tubular processes, including renal tubular fibrosis, urinary DKK3 could be a marker of renal tubular stress and/or renal tubular tissue at risk^9,10,14,15^. In this study, in *blood plasma* we also observed a strong correlation between DKK3 and kidney function, but DKK3 was not independently associated with both prevalent and new-onset CKD. This is probably explained by the fact that, though DKK3 is produced in several organs and tissues^16,22^, circulating levels of DKK3 are likely a reflection of production in all of these tissues, whilst on the other hand, urinary DKK3 will inevitably predominantly originate from tubular epithelium, and is thereby much more kidney specific^9,10^. In line with the suggestion that organ/tissue specificity of a biomarker of disease is an important factor^23,24^, this could potentially explain why urinary DKK3 is highly predictive for future loss of kidney function, and likewise explains why this is not the case for plasma DKK3. This is further supported by the fact that in CKD patients urinary DKK3 levels strongly correlate with UAE^14^, whilst in this study only a minor correlation between plasma DKK3 and UAE was observed, indicating that urinary DKK3, but not plasma DKK3, is associated with kidney damage. Thus, in the general population as a whole, *blood plasma* DKK3 seems not a suitable predictive marker for CKD. Interestingly, in subjects with no kidney disease at baseline, in those with the lowest UAE-values (according to UAE-tertiles) DKK3 *blood plasma* levels did independently predict for new-onset CKD, even when correct for a multivariable model including eGFR. This suggests that in subjects in which kidney damage is close to absent, blood plasma DKK3 can add to CKD risk prediction.

In a recent study performed by Yu et al. it was shown that DKK3 *blood plasma* levels are inversely associated with BMI, alongside with an inverse relationship between DKK3 plasma levels and atherosclerosis^13^, suggesting DKK3 could be a biomarker of endothelial status. Multiple studies described the role of DKK3 in atherosclerosis development using atherosclerosis-prone apolipoprotein E-deficient (ApoE^−/−^) mice, but these studies showed opposing results, either suggesting accelerated or reduced atherosclerosis in DKK3^−/−^ mice^11–13^. In our study, we investigated the relationship between DKK3 and both prevalent and new-onset CVD. In this general population cohort, which was larger and therewith better powered than the study of Yu et al., after correction for common risk factors and clinical variables, we did not find an independent relationship between DKK3 and CVD. Possibly, since the cohort used in this study is on average younger than in the study of Yu et al., and DKK3 is strongly correlated with age^25^, this could partly explain these differences. The (cardiovascular risk) factors included in the multivariable model as described in this study explained approximately 18% of the variance in DKK3 plasma levels and similar variables as described before were included in this model, including age, BMI and estimated glomerular filtration rate (eGFR)^16^. However, the variables and circumstances that regulate the remainder 82% of variance in DKK3 remains unexplained. Possibly, these include genetic variations, inflammatory status and/or other characteristics not encountered for in this study. Recent evidence indicates that the extracellular matrix serine protease, HtrA1, can target DKK3 for degradation and may regulate its levels, at least in aqueous humor of the eye^26^. The role of HtrA1 in controlling plasma DKK3 protein levels is unknown, but deserves attention in future investigations. Nevertheless, together with the observations of Yu et al.^13^, this study confirms a link between DKK3 and cardiovascular risk factors.

Some limitations of this study should be mentioned. Firstly, the study sample for the PREVEND cohort was derived from general population of Groningen, the Netherlands, which might limit the generalization of our results towards the global population. However, its large cohort size, the long follow-up period and its specific design to study CVD and CKD over time makes this cohort an ideal choice to answer our research questions. Secondly, due to its design and study setup, subjects with albuminuria were overrepresented in this cohort. By applying statistical weights we aimed to make this research more applicable to the general population. Finally, this research was performed in a general population cohort consisting of, predominantly, healthy subjects. Possibly, studies in specific disease cohorts, for example cohorts with patients diagnosed with renal failure, could provide more insight into DKK3 and its association with kidney disease.

## Conclusion

Together, this study shows that DKK3 plasma levels are associated with several cardiovascular risk factors, including age, BMI, glucose levels and sex. However, DKK3 plasma levels are generally not independently associated with prevalent and new-onset CVD and CKD and might only have predictive value for new-onset CKD in those subjects with the lowest UAE values.

## Methods

### Study population

For this study, the PREVEND cohort was used, which is described in detail elsewhere^17^. In short, the PREVEND cohort was designed as a prospective study to investigate the natural course of microalbuminuria, as well to study the association between microalbuminuria and new-onset renal diseases and CVD. A questionnaire regarding demographics, cardiovascular history and renal history was send to subjects from the general population aged 28–75 years, accompanied by a vial to collect a morning urine sample. A response was received from a total of 40856 out of 85421 invited subjects. Pregnant subjects and subjects using insulin were excluded. Next, all subjects with a morning urinary albumin concentration (UAC) > 10 mg/L and a random selection of subjects with UAC < 10 mg/L were included, resulting in a total of 8592 subjects. These subjects made two visits to an outpatient clinic, where several clinical examinations were performed and two 24 h urine samples and blood samples were collected. Ethylenediaminetetraacetic acid (EDTA) plasma samples were stored at − 80 °C until analysis. The 172 subjects for which plasma samples were not available for the measurement of DKK3 were excluded from the analysis, resulting in a final study cohort of 8420 subjects. A flow chart with a detailed description of the selection procedure is shown in Supplemental Figure S1, also including an overview of the number of prevalent and new-onset CVD and CKD cases.

### Measurement of DKK3

Enzyme-linked immunosorbent assays (ELISA, DY1118, R&D systems, USA) combined with half area 96 wells plates (3690, Costar half area 96 well polystyrene assay plates, Corning, USA) were used to measure DKK3 plasma levels. Standard curves were included in duplicate on each plate. Plasma samples were diluted 1:100 using 1% bovine serum albumin (BSA, 11930.03, Serva, Germany) in phosphate buffered saline (PBS) as a dilution buffer. PBS with 0.05% Tween (Tween 20, P1379, Sigma-Aldrich, USA) was used as a wash buffer. TMB (Tetramethylbenzidine-dihydrochloride, T3405, Sigma-Aldrich, USA), H_2_O_2_ (Hydrogen peroxide 30%, Emsure, 1.07209.0250, VWR, USA), ultra-purified H_2_O and NaAc (Sodiumacetate, S2889, Sigma-Aldrich, USA) were combined to form the substrate solution and 2 N H_2_SO_4_ (Sulfuric acid, 7102, Avantor, USA) was used as the stop solution. Concentrations were determined using a spectrophotometer (Synergy H1 microplate spectrophotometer, BioTek, USA). Before measuring the total cohort, we performed quality control and determined the ideal sample dilution factor. This has been described in detail recently^16^. Importantly, the coefficient of variance the assay was 8.9%, the inter-assay reproducibility was 97.2% (uncorrected for the coefficient of variance), and the stability of DKK3 over four freeze–thaw cycles was 7.7% (uncorrected for the coefficient of variance).

### Laboratory measurements and definitions

As previously described, urinary albumin concentration was determined using nephelometry (Dade Behring Diagnostics, Germany) at inclusion^18^. The mean urinary albumin excretion (UAE) of the two 24 h urine excretions collected at baseline was used as the UAE value. Plasma glucose, serum cholesterol and serum creatinine levels were determined by Kodak Ektachem dry chemistry (Eastman Kodak, USA)^18^. Body mass index (BMI) was calculated as the ratio between weight in kilograms and the square of height in meters. Diabetes mellitus was defined as fasting blood glucose levels ≥ 7.8 mmol/L, non-fasting glucose ≥ 11.1 mmol/L and/or the use of antidiabetic drugs. Hypertension was defined as a diastolic blood pressure (DBP) ≥ 90 mmHg, a systolic blood pressure (SBP) ≥ 140 mmHg and/or the use of anti-hypertensive treatment. Estimated glomerular filtration rate (eGFR) was calculated using the combined creatinine cystatin C-based Chronic Kidney Disease Epidemiology (CKD-EPI) Collaboration equation from 2012^19^. Prevalent cardiovascular disease (CVD) was defined as a subject-reported positive history of myocardial infarction and/or cerebrovascular accident. New-onset CVD was defined as having an event of myocardial infarction, acute or subacute ischemic heart disease, subarachnoid or intracerebral hemorrhage or ischemic stroke, or the need to undergo coronary artery bypass grafting (CABG) or percutaneous coronary intervention (PCI) during the follow-up period. Prevalent and new-onset chronic kidney disease (CKD) were, according to the KDIGO criteria, defined as an eGFR ˂ 60 ml/min/1.73m2 or a UAE > 30 mg/24 h at baseline or during follow-up, respectively^20^.

### Statistical analyses

Normality of data was assessed using histograms with fitted normal curves, QQ-plots, Shapiro–Wilk tests, skewness values and kurtosis values. Normally distributed continuous variables are presented as means ± standard deviation (SD), non-normally distributed continuous variables as medians with interquartile range (IQR), and categorical data as n (%). P-for trend over quintiles of DKK3 was calculated using linear regression analysis for normally distributed variables, the Mann–Kendall test for non-normally distributed variables and the Cochran-Armitage trend test for categorical variables. For the further statistical analyses, non-normally distributed variables, including DKK3 values, were log2-transformed. Univariate regression analysis was performed and results are displayed as standardized betas (Sβ) and R-square (R^2^). For a selection of the strongest correlates in univariate regression analysis, the association with DKK3 was further explored using fractional polynomial prediction plots with 95% confidence intervals (CI). Variables showing a P-value ˂ 0.10 in univariate regression analysis were included in multivariable regression analysis and variables were included using forward selection. In linear regression analysis, normality and homogeneity of residuals were tested using PP-plots and QQ-plots, respectively. The presence of multicollinearity was checked using variance inflation factors. To investigate the association of DKK3 with prevalent CVD and CKD, logistic regression analysis was performed and results are displayed as odds ratios (OR) with 95% CI. The association of baseline DKK3 with new-onset CVD and CKD was tested using Cox proportional hazards regression analysis and results are displayed as hazard ratios (HR) with 95% CI. Assumptions for proportional hazards in Cox proportional hazards regression were tested using Schoenfeld residuals. Logistic regression analysis and Cox proportional hazards regression analysis were performed crude, age-adjusted, adjusted for age and sex, corrected for a multivariable model as composed by multivariable linear regression analysis (model includes the variables age, eGFR, BMI, CRP, glucose and sex) (Table 3), and corrected for clinical relevant variables referred to as clinical model, including age, sex, BMI, systolic blood pressure, glucose and cholesterol. When analyzing for CVD, eGFR was also included in this clinical model. Subjects with CKD or CVD at baseline were excluded when analyzing for new-onset CKD or CVD, respectively. Cox proportional hazards regression analyses were performed for the whole cohort and for subgroups based on eGFR tertiles and UAE tertiles. Fine-Gray competing risk regression models with death as the competing risk were used to correct for fatal events during follow-up when assessing the association between baseline DKK3 and new-onset CVD and new-onset CKD. Subjects with albuminuria were overrepresented in this cohort. Therefore, using a weighted Cox regression model a statistical correction factor was applied to make this research more applicable to the general population, and this was performed as described before^21^. A P-value of ˂ 0.05 was considered statistically significant. All statistical analyses were performed using Stata version 14 (Stata Corporation, College Station, USA).

### Ethics declarations

The PREVEND study was performed in accordance with the guidelines of the declaration of Helsinki and was approved by the medical ethics committee of the University Medical Center Groningen (UMCG). Written informed consent was received from all participants.

## Supplementary Information

Supplementary Information 1.

Supplementary Information 2.

## Data Availability

Please contact the authors for data requests.
