# Supplementary material for: The emerging plasma biomarker Dickkopf-3 (DKK3) and its association with renal and cardiovascular disease in the general population
Source: Sci Rep. 2021 Apr 21;11:8642. doi: 10.1038/s41598-021-88107-9 (PMC8060267; doi:10.1038/s41598-021-88107-9)
Supplement: Supplementary file 1 — Supplementary Information 1. [file 41598_2021_88107_MOESM1_ESM.pdf]

SUPPLEMENTANTARY FIGURE

To the manuscript entitled:

The emerging plasma biomarker Dickkopf-3 (DKK3) and its association with renal and cardiovascular disease in the general population

Arnold Piek[1], Leonie Smit[1], Navin Suthahar[1], Stephan J.L. Bakker[2], Rudolf A. de Boer[1], Herman H.W. Silljé[1]

[1] Department of Cardiology, University Medical Center Groningen, University of Groningen, The Netherlands

[2] Department of Internal Medicine, University Medical Center Groningen, University of Groningen, The Netherlands

*Corresponding author:

H.H.W. Silljé, PhD

Department of Cardiology, University Medical Center Groningen

University of Groningen

Hanzeplein 1, 9713 GZ Groningen, The Netherlands

P.O. Box 30.001, 9700 RB Groningen, The Netherlands

Phone: +31 50 361 5523, fax: +31 50 3611347, email: h.h.w.sillje@umcg.nl

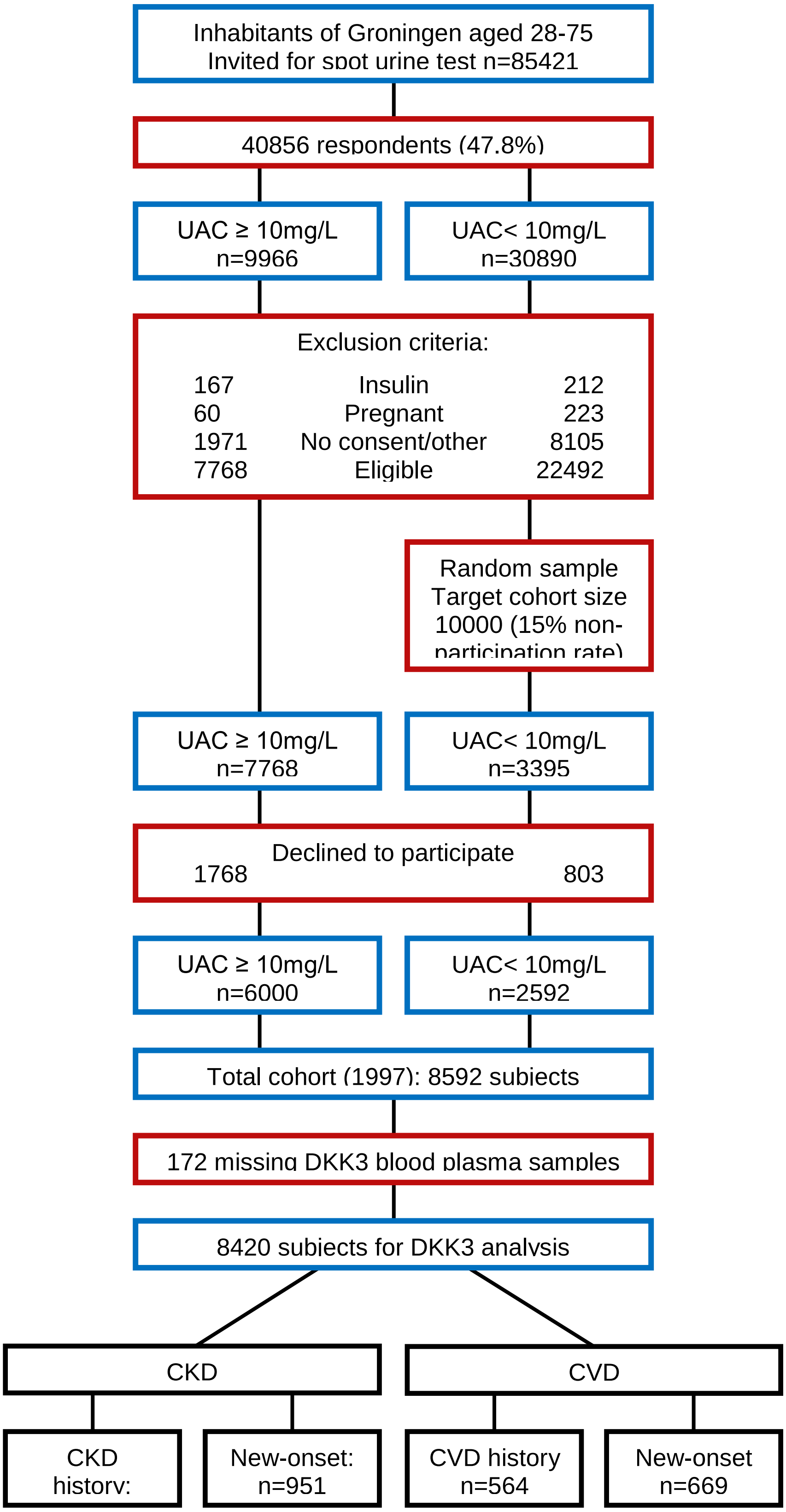

Supplementary Figure 1. Schematic depiction of the study subject selection procedure.

A total of 85421 subjects aged 28-75 years were invited to participate in this cohort study. A

response was received from a total of 40856 subjects. Of these, 9966 subjects had a morning urinary albumin concentration (UAC) > 10 mg/L and 30890 an UAE < 10 mg/L. Exclusion criteria were insulin usage, current pregnancy or no consent. A random sample was drawn from the subpopulation with an UAC < 10 mg/L. Hereafter, a small group of subjects declined to participate. The final cohort consisted of 8592 subjects, of which 6000 had an UAC > 10 mg/L and 2592 an UAC < 10 mg/L. A total of 172 plasma samples were unavailable for baseline Dickkopf-3 (DKK3) plasma measurement, thus 8420 subjects DKK3 plasma concentrations were measured. At baseline, 1361 subjects had a history of chronic kidney disease (CKD) and 564 had a history of cardiovascular disease (CVD). During follow-up, new-onset CKD was observed in 951 subjects and new-onset CVD in 669 subjects.
