# Supplementary material for: The emerging plasma biomarker Dickkopf-3 (DKK3) and its association with renal and cardiovascular disease in the general population
Source: Sci Rep. 2021 Apr 21;11:8642. doi: 10.1038/s41598-021-88107-9 (PMC8060267; doi:10.1038/s41598-021-88107-9)
Supplement: Supplementary file 2 — Supplementary Information 2. [file 41598_2021_88107_MOESM2_ESM.pdf]

SUPPLEMENTANTARY TABLES

To the manuscript entitled:

The emerging plasma biomarker Dickkopf-3 (DKK3) and its association with renal and cardiovascular disease in the general population

Arnold Piek[1], Leonie Smit[1], Navin Suthahar[1], Stephan J.L. Bakker[2], Rudolf A. de Boer[1], Herman H.W. Silljé[1]

[1] Department of Cardiology, University Medical Center Groningen, University of Groningen, The Netherlands

[2] Department of Internal Medicine, University Medical Center Groningen, University of Groningen, The Netherlands

*Corresponding author:

H.H.W. Silljé, PhD

Department of Cardiology, University Medical Center Groningen

University of Groningen

Hanzeplein 1, 9713 GZ Groningen, The Netherlands

P.O. Box 30.001, 9700 RB Groningen, The Netherlands

Phone: +31 50 361 5523, fax: +31 50 3611347, email: h.h.w.sillje@umcg.nl

Table S1. DKK3: New-onset CVD with death as a competing risk

	Hazard ratio (95% CI)	P-value
DKK3[†]	1.45 (1.12 – 1.88)	0.005
DKK3[†], age	0.78 (0.56 – 1.07)	0.126
DKK3[†], age, sex	N.A.	N.A.
DKK3[†], models		
Multivariable model	N.A.	N.A.
Clinical model	N.A.	N.A.

CI=Confidence Interval. CVD=Cardiovascular disease. DKK3=Dickkopf-3. Multivariable model as presented in table 3. Clinical model includes age, sex, body mass index, systolic blood pressure, glucose[†], cholesterol and estimated glomerular filtration rate[†]. N=7828. [†]Log2-transformed.

Table S2. DKK3: Prevalent and new-onset CVD per eGFR tertile

Baseline eGFR Tertiles, mL/min/1.73m²	New-onset CVD		
	**Cases, n (%)**	**Odds ratio (95% CI)**	**P-value**
**Q1: eGFR < 89.8 (N=2453)**			
DKK3†	374 (15.2)	1.69 (1.16-2.47)	0.006
DKK3†, age	374 (15.2)	1.21 (0.79-1.85)	0.366
DKK3†, age, sex	N.A.	N.A.	N.A.
DKK3†, models			
Multivariable model	N.A.	N.A.	N.A.
Clinical model	N.A.	N.A.	N.A.
**Q2: eGFR 89.8-104.4 (N=2453)**			
DKK3†	154 (6.3)	0.74 (0.45-1.23)	0.253
DKK3†, age	N.A.	N.A.	N.A.
DKK3†, age, sex	N.A.	N.A.	N.A.
DKK3†, models			
Multivariable model	N.A.	N.A.	N.A.
Clinical model	N.A.	N.A.	N.A.
**Q3: eGFR > 104.4 (N=2453)**			
DKK3†	82 (3.3)	0.57 (0.19-1.71)	0.314
DKK3†, age	N.A.	N.A.	N.A.
DKK3†, age, sex	N.A.	N.A.	N.A.
DKK3†, models			
Multivariable model	N.A.	N.A.	N.A.
Clinical model	N.A.	N.A.	N.A.

CI=Confidence Interval. CVD=Cardiovascular disease. DKK3=Dickkopf-3. eGFR=Estimated glomerular filtration rate. Multivariable model as presented in Table 3. Clinical model includes age, sex, body mass index, systolic blood pressure, glucose†, cholesterol and eGFR. †Log2-transformed.

Table S3. DKK3: Prevalent and new-onset CVD per UAE tertile

**Baseline UAE Tertiles, mg/24h**	**New-onset CVD**		
	**Cases, n (%)**	**Odds ratio (95% CI)**	**P-value**
**Q1: UAE <7.1 (N=2613)**			
DKK3†	120 (4.6)	1.20 (0.76-1.92)	0.433
DKK3†, age	N.A.	N.A.	N.A.
DKK3†, age, sex	N.A.	N.A.	N.A.
DKK3†, models			
Multivariable model	N.A.	N.A.	N.A.
Clinical model	N.A.	N.A.	N.A.
**Q2: UAE 7.1-13.0 (N=2611)**			
DKK3†	183 (7.0)	1.73 (0.96-3.09)	0.068
DKK3†, age	N.A.	N.A.	N.A.
DKK3†, age, sex	N.A.	N.A.	N.A.
DKK3†, models			
Multivariable model	N.A.	N.A.	N.A.
Clinical model	N.A.	N.A.	N.A.
**Q3: UAE >13.0 (N=2607)**			
DKK3†	366 (14.0)	1.89 (1.34-2.66)	< 0.000
DKK3†, age	366 (14.0)	1.03 (0.69-1.53)	0.901
DKK3†, age, sex	N.A.	N.A.	N.A.
DKK3†, models			
Multivariable model	N.A.	N.A.	N.A.
Clinical model	N.A.	N.A.	N.A.

CI=Confidence Interval. CVD=Cardiovascular disease. DKK3=Dickkopf-3. UAE=Urinary albumin excretion. Multivariable model as presented in Table 3. Clinical model includes age, sex, body mass index, systolic blood pressure, glucose†, cholesterol and eGFR. †Log2-transformed.

Table S4. DKK3: New-onset CKD with death as a competing risk

	Hazard ratio (95% CI)	P-value
DKK3†	1.67 (1.37 – 2.03)	< 0.001
DKK3†, age	1.03 (0.81 – 1.32)	0.786
DKK3†, age, sex	N.A.	N.A.
DKK3†, models		
Multivariable model	N.A.	N.A.
Clinical model	N.A.	N.A.

CI=Confidence Interval. CKD=Chronic kidney disease. DKK3=Dickkopf-3. Multivariable model as presented in table 3. Clinical model includes age, sex, body mass index, systolic blood pressure, glucose† and cholesterol†. N=5548. †Log2-transformed.

Table S5. DKK3: Prevalent and new-onset CKD per eGFR tertile

Baseline eGFR Tertiles mL/min/1.73m^2	New-onset CVD		
	**Cases, n (%)**	**Odds ratio (95% CI)**	**P-value**
**Q1: eGFR 60.1-91.2 (N=1810)**			
DKK3†	488 (27.0)	1.93 (1.42-2.61)	< 0.001
DKK3†, age	488 (27.0)	1.22 (0.85-1.75)	0.275
DKK3†, age, sex	N.A.	N.A.	N.A.
DKK3†, models			
Multivariable model	N.A.	N.A.	N.A.
Clinical model	N.A.	N.A.	N.A.
**Q2: eGFR 91.2-105.1 (N=1810)**			
DKK3†	239 (13.2)	0.90 (0.60-1.34)	0.590
DKK3†, age	N.A.	N.A.	N.A.
DKK3†, age, sex	N.A.	N.A.	N.A.
DKK3†, models			
Multivariable model	N.A.	N.A.	N.A.
Clinical model	N.A.	N.A.	N.A.
**Q3: eGFR >105.2 (N=1810)**			
DKK3†	166 (9.2)	0.89 (0.54-1.46)	0.641
DKK3†, age	N.A.	N.A.	N.A.
DKK3†, age, sex	N.A.	N.A.	N.A.
DKK3†, models			
Multivariable model	N.A.	N.A.	N.A.
Clinical model	N.A.	N.A.	N.A.

CI=Confidence Interval. CVD=Cardiovascular disease. DKK3=Dickkopf-3. eGFR=Estimated glomerular filtration rate. Multivariable model as presented in Table 3. Clinical model includes age, sex, body mass index, systolic blood pressure, glucose† and cholesterol. †Log2-transformed.

Table S6. DKK3: Prevalent and new-onset CKD per UAE tertile

**Baseline UAE Tertiles mg/24h**	**New-onset CVD**		
	**Cases, n (%)**	**Odds ratio (95% CI)**	**P-value**
**Q1: UAE <6.6 (N=1857)**			
DKK3†	142 (7.6)	2.01 (1.50-2.70)	< 0.001
DKK3†, age	142 (7.6)	1.67 (1.17-2.38)	0.005
DKK3†, age, sex	142 (7.6)	1.68 (1.18-2.40)	0.004
DKK3†, models			
Multivariable model	130 (7.4)	1.89 (1.22-2.92)	0.004
Clinical model	139 (7.5)	1.82 (1.30-2.56)	0.001
**Q2: UAE 6.6-10.5 (N=1842)**			
DKK3†	190 (10.3)	1.90 (1.21-2.96)	0.005
DKK3†, age	190 (10.3)	0.69 (0.40-1.18)	0.175
DKK3†, age, sex	N.A.	N.A.	N.A.
DKK3†, models			
Multivariable model	N.A.	N.A.	N.A.
Clinical model	N.A.	N.A.	N.A.
**Q3: UAE >10.6 (N=1849)**			
DKK3†	619 (33.5)	1.35 (0.97-1.86)	0.072
DKK3†, age	N.A.	N.A.	N.A.
DKK3†, age, sex	N.A.	N.A.	N.A.
DKK3†, models			
Multivariable model	N.A.	N.A.	N.A.
Clinical model	N.A.	N.A.	N.A.

CI=Confidence Interval. CVD=Cardiovascular disease. DKK3=Dickkopf-3. UAE=Urinary albumin excretion. Multivariable model as presented in Table 3. Clinical model includes age, sex, body mass index, systolic blood pressure, glucose† and cholesterol. †Log2-transformed.
